# Renal Denervation in a Patient With Resistant Hypertension Using a Second-Generation Multi-electrode Catheter in Puerto Rico: A Non-pharmacological Technique for Resistant Arterial Hypertension

**DOI:** 10.7759/cureus.105021

**Published:** 2026-03-11

**Authors:** Juan F Rodríguez Acosta, Frantony Mercado Cabrera, Héctor Martínez González, Joseph Maldonado Suárez, Brian Torres Mercado, Xavier Delgado, Daniela Henao Escobar, David R Bermúdez Núñez

**Affiliations:** 1 Cardiology/Interventional Cardiology, Cardiovascular Diseases Fellowship Program, Mayagüez Medical Center, Mayagüez, PRI; 2 Cardiology, Cardiovascular Diseases Fellowship Program, Mayagüez Medical Center, Mayagüez, PRI; 3 Medical School, Centro Médico Episcopal San Lucas, Ponce, PRI; 4 School of Medicine, Ponce Health Sciences University, Ponce, PRI

**Keywords:** cardiac mibi scan, drug-resistant hypertension, non-pharmacological technique, renal nerve denervation, spyral catheter

## Abstract

Resistant arterial hypertension poses a clinical challenge, often requiring novel interventions beyond pharmacologic therapy. Renal denervation (RDN), a catheter-based technique targeting renal sympathetic nerves, has emerged as a promising adjunctive treatment.

A 44-year-old female with resistant hypertension, unresponsive to triple antihypertensive therapy (clonidine 0.2 mg orally twice a day, losartan 100 mg orally daily, and hydrochlorothiazide 25 mg orally daily), presented with hypertensive urgency (blood pressure: 180/109 mmHg). Secondary causes of hypertension were excluded. She underwent bilateral renal denervation using the Symplicity Spyral second-generation radiofrequency catheter (Medtronic, Minneapolis, MN, US).

Given persistently elevated blood pressure despite guideline-directed medical therapy and no secondary etiology, the multidisciplinary team opted for RDN. The procedure was performed via right femoral arterial access. A total of 33 ablations were applied (22 on the right renal artery, 11 on the left renal artery) with no procedural complications.

This case represents the first documented RDN in Puerto Rico using a second-generation multi-electrode system. The patient achieved blood pressure control by day 3 post-procedure, maintained at the three-month follow-up without major medication changes or adverse events. RDN offers a safe, effective, and minimally invasive strategy for managing resistant hypertension in selected patients.

## Introduction

Resistant arterial hypertension represents a significant clinical challenge, traditionally defined as blood pressure that remains above target despite the combined use of three antihypertensive agents of different classes at adequate doses. Although ambulatory blood pressure monitoring is the preferred method to confirm true resistance, it is not always available in all clinical settings. In this context, renal denervation has emerged as a minimally invasive therapeutic alternative designed to modulate renal sympathetic nervous system activity, a key factor in hypertension pathophysiology [1,2]. This procedure, performed via radiofrequency or ultrasound ablation techniques, aims to disrupt afferent and efferent sympathetic impulses at the level of the renal arteries [3,4]. Our case describes a 44‑year‑old woman who presented with hypertensive urgency while already receiving optimal doses of triple antihypertensive therapy: clonidine 0.2 mg orally twice a day, losartan 100 mg orally daily, and hydrochlorothiazide 25 mg orally daily. Our case also represents the first renal denervation procedure performed in Puerto Rico, highlighting clinical indications, emphasizing technical aspects, sharing procedural results, and underscoring its relevance as an advancement in the management of patients with difficult‑to‑control hypertension.

## Case presentation

The procedure was performed on a 44‑year‑old woman with a medical history of bronchial asthma and resistant arterial hypertension on clonidine 0.2 mg orally twice a day, losartan 100 mg orally daily, and hydrochlorothiazide 25 mg orally daily. She was admitted with severely elevated blood pressure consistent with hypertensive urgency, with a baseline blood pressure of 180/109 mmHg, heart rate of 98 bpm, respiratory rate of 18 breaths per minute, temperature of 37.0 °C, and oxygen saturation of 96% on room air, without evidence of target organ damage (TOD).

Evaluation for secondary causes of hypertension was guided by the patient’s history and the clinical information available during admission. A comprehensive evaluation, including abdominal-pelvic CT, renal ultrasound, myocardial perfusion imaging (MPI), transthoracic echocardiography (TTE), renal Doppler studies, and biochemical testing were done and did not point to secondary causes of hypertension. However, conditions such as obstructive sleep apnea were not evaluated during the admission, as the patient’s history did not suggest this etiology as a possible culprit.

The patient's laboratory values are summarized in Table 1.

**Table 1:** Patients' laboratory data PT: prothrombin time; aPTT: activated partial thromboplastin time; INR: international normalized ratio; BUN: blood urea nitrogen; eGFR: estimated glomerular filtration rate; AST: aspartate aminotransferase; ALT: alanine transaminase; CRP: C-reactive protein; TSH: thyroid-stimulating hormone

Test	Your Value	Reference Range (Adult)
WBC	7.74 ×10³/µL	4.0–11.0 ×10³/µL
RBC	4.59 ×10⁶/µL	4.0–5.5 ×10⁶/µL
Hemoglobin	12.8 g/dL	12.0–16.0 g/dL
Hematocrit	38.50%	36–48%
PT	14.0 s	11–15 s
aPTT	32.3 s	25–35 s
INR	1.17	0.8–1.2
Sodium	138 mmol/L	135–145 mmol/L
Potassium	4.2 mmol/L	3.5–5.0 mmol/L
Chloride	102 mmol/L	98–107 mmol/L
CO₂ (Bicarbonate)	23 mmol/L	22–29 mmol/L
Anion Gap	17 mmol/L	8–16 mmol/L (lab‑dependent)
BUN	10.9 mg/dL	7–20 mg/dL
Creatinine	0.70 mg/dL	0.6–1.3 mg/dL
eGFR	110 mL/min	≥90 mL/min
Glucose	166 mg/dL	70–99 mg/dL (fasting)
Osmolality	278 mOsm/kg	275–295 mOsm/kg
Calcium	8.8 mg/dL	8.6–10.2 mg/dL
AST	34 U/L	10–40 U/L
ALT	45 U/L	7–56 U/L
hs‑Troponin T	<6 ng/L	≤14 ng/L
CRP	1.1 mg/L	<3 mg/L
NT‑proBNP	90 pg/mL	<125 pg/mL
Albumin	4.3 g/dL	3.5–5.0 g/dL
Globulin	2.6 g/dL	2.0–3.5 g/dL
TSH	3.84 µIU/mL	0.4–4.0 µIU/mL
Urine Protein	Negative	Negative
Urine Blood	Negative	Negative
Urine Ketones	Negative	Negative
Specific Gravity	1.018	1.005–1.030
Pregnancy Test	Negative	Negative

It is worth noting that the elevated glucose level (166 mg/dL) was interpreted in the context of the patient's presentation to the emergency department. The timing of the patient’s last meal was not known, and acute severe hypertension can produce transient stress‑related hyperglycemia. Therefore, it is unclear whether this reflected recent food intake, a stress response, a combination, or an underlying metabolic abnormality; however, her history, physical examination, and review of systems did not suggest chronic hyperglycemia.

Given persistent severe hypertension despite guideline‑directed medical therapy and the absence of a reversible etiology, the multidisciplinary team proceeded with renal denervation using the Symplicity Spyral System (Medtronic, Minneapolis, MN, US). This minimally invasive, catheter‑based technique targets renal sympathetic nerve activity through radiofrequency ablation, aiming to achieve durable blood pressure reduction in patients with resistant hypertension.

Procedure summary: Symplicity Spyral System (Medtronic)

This is a minimally invasive, catheter‑based procedure designed to reduce renal sympathetic nerve activity, aiming to lower blood pressure in resistant hypertension patients [5].

A detailed description of our procedure is as follows: A renal sympathetic denervation procedure was performed after obtaining informed consent from the patient. Then, under conscious sedation, a right percutaneous femoral arterial access was obtained using a 6Fr sheath, followed by the advancement of a 6Fr Launcher IMA 90 cm guide catheter (Medtronic) and a 0.014″ × 190 cm HI‑TORQUE Iron Man guidewire (Abbott Cardiovascular, Abbott Park, IL, US) into the renal vasculature. Intravenous heparin 15,000 IU was administered for anticoagulation. Each renal artery received 200 µg of intrarenal Tridil to optimize vessel caliber. A diagnostic renal angiogram of both renal arteries was performed and met the anatomical criteria for renal denervation, with both left and right main renal artery length being ≥20 mm, diameters ≥4 mm, no evidence of stenosis, no fibromuscular dysplasia, and no early bifurcation [6]. 

**Figure 1:**
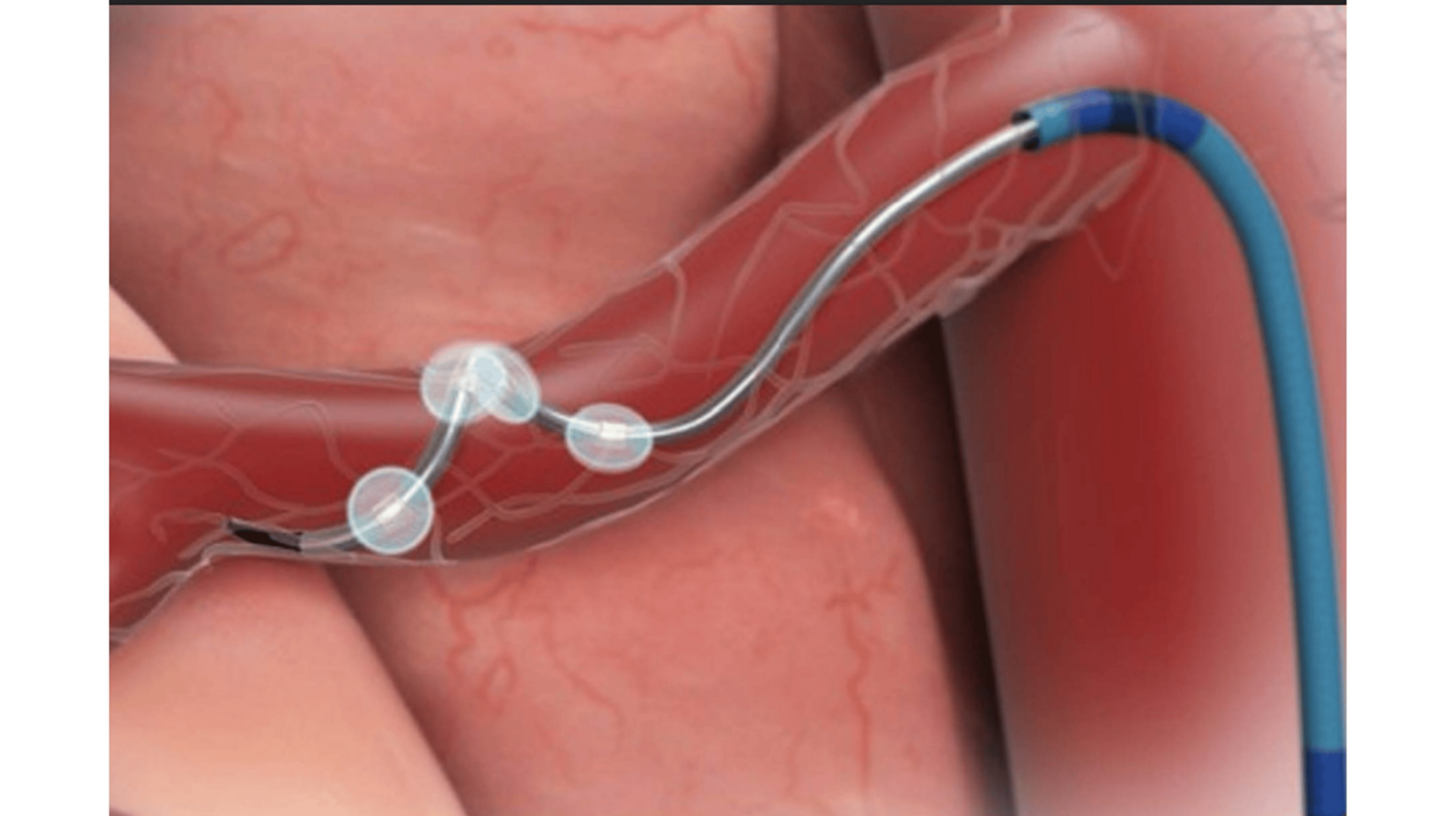
Symplicity Spyral catheter Image reproduced with permission from the original owner, Medtronic (Minneapolis, MN, US).

**Figure 2:**
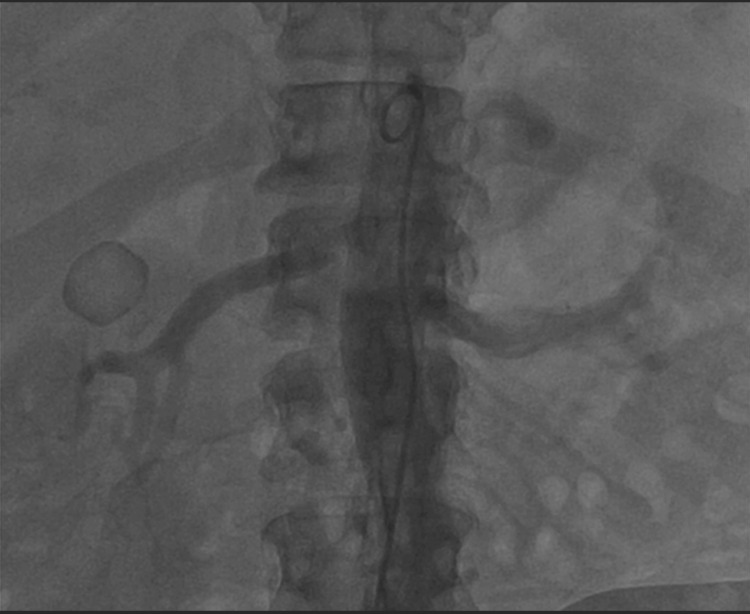
Abdominal arterial arteriography showing the main renal arteries' diameters: ~7 mm (left renal artery), ~6 mm (right renal artery) Adequate catheter access was achieved.

**Figure 3:**
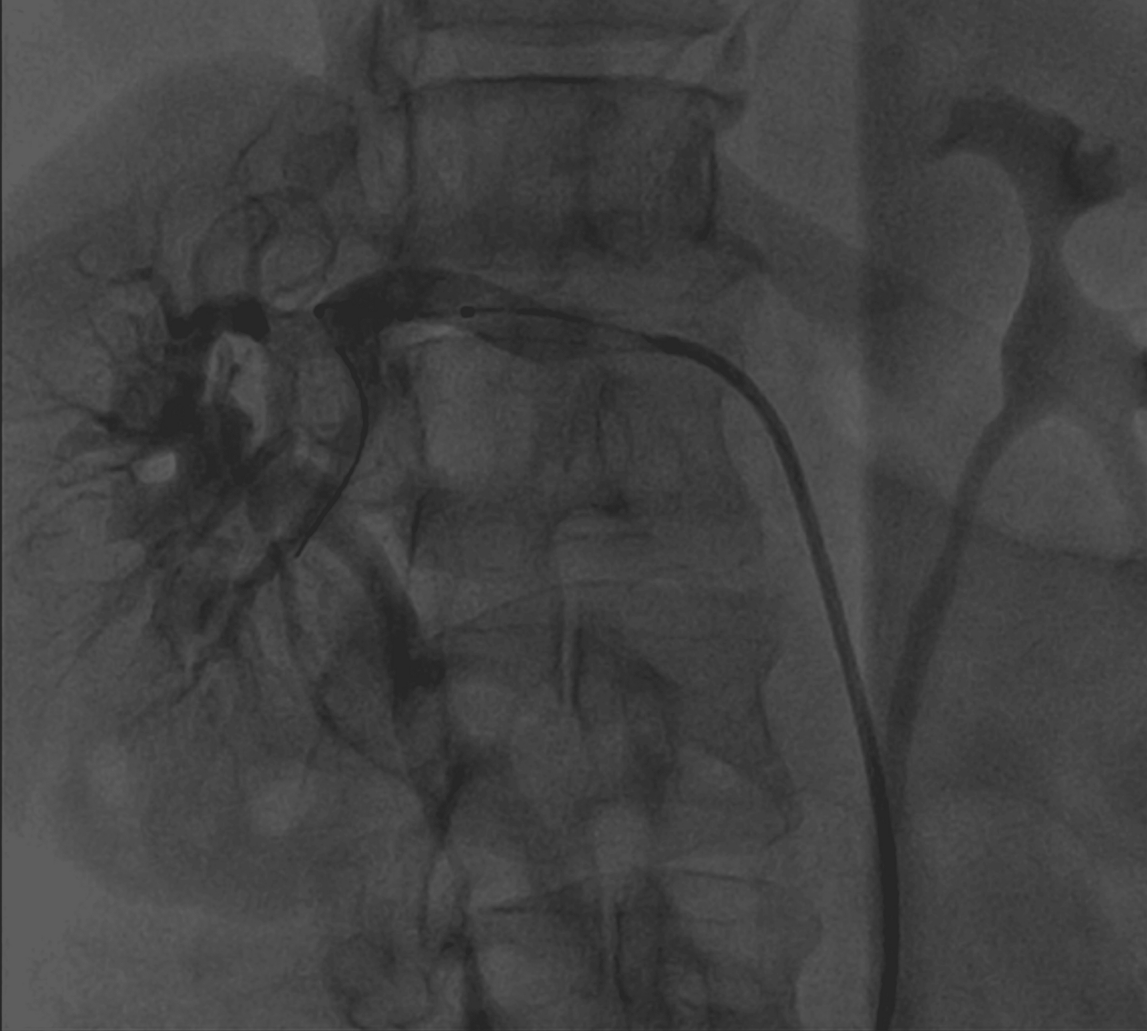
Right renal arterial arteriography showing the Symplycity Spyral RDN catheter (22 ablations performed; 11 ablations were performed on the left renal artery; not shown) RDN: renal denervation

The Symplicity Spyral catheter (Medtronic), featuring four spiral‑arranged electrodes, was advanced over the guidewire and positioned sequentially within each renal artery (Figures 1-3). Medium‑frequency radiofrequency energy (approximately 460 kHz) was delivered to create controlled periadventitial sympathetic nerve ablation while preserving arterial integrity, with multiple helical ablation cycles lasting approximately 60 seconds to ensure circumferential sympathetic denervation. Upon completion, the catheter system was withdrawn, the femoral access site was closed with a Perclose, and the patient was monitored during an uncomplicated recovery period lasting under one hour.

Electrode temperature, impedance, and impedance drop remained within expected ranges. Occasional generator codes indicating suboptimal electrode contact were addressed by re-applying energy to specific electrodes.

 Patient evolution

**Figure 4:**
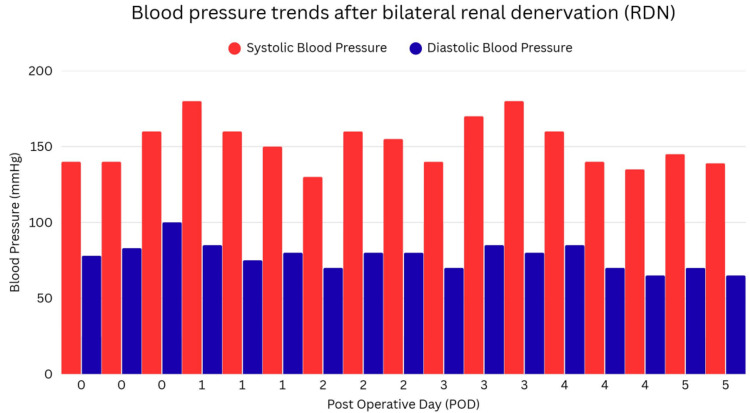
Serial monitoring of the patients' blood pressure following the procedure

From post-procedure day 3, the patient's blood pressure trends averaged at 130/90 mmHg, and she remained clinically stable with preserved renal function, afebrile, and no vascular complications (Figure 4). Although this marked a meaningful reduction from her pre‑procedure blood pressure trends, the average blood pressure trended above the American Heart Association (AHA)/American College of Cardiology (ACC) guideline target of <130/80 mmHg, and the diastolic value remained within the stage 2 hypertension range.

More importantly, during the subsequent two months of outpatient follow‑up, her blood pressure remained stable and lower than baseline without any adjustments to antihypertensive therapy, no cardiovascular events, and no adverse symptoms.

## Discussion

Hypertension is a leading but modifiable risk factor for cardiovascular morbidity and mortality. Despite broad therapeutic availability, global blood pressure control rates continue to decline, and in the United States, only ~23 % of adults with hypertension achieve guideline‑recommended targets. The 2025 AHA/ACC/Multisociety High Blood Pressure Guideline maintains the established classification of blood pressure categories and treatment goals, as summarized in Table 2. These definitions continue to guide clinical decision‑making and reflect evidence from contemporary intensive treat‑to‑target trials [7]. In 2023, the FDA approved RDN as an adjunctive catheter‑based therapy for patients whose blood pressure remains uncontrolled despite lifestyle modification and antihypertensive medications [8]. This approval followed multiple rigorously designed randomized, sham‑controlled trials demonstrating modest but statistically significant reductions in blood pressure across a spectrum of hypertensive severities. Individual variability in response underscores the need for continued research to refine patient selection and identify those most likely to benefit. Early safety data remain favorable, and ongoing studies are evaluating long‑term efficacy and durability. From a historical standpoint, the first RDN procedure in Puerto Rico was performed in September 2024, representing a key milestone in the adoption of this therapy [9].

**Table 2:** 2025 AHA/ACC blood pressure categories and treatment goals AHA: American Heart Association; ACC: American College of Cardiology Source: [10]

Category	Systolic BP (mmHg)	Diastolic BP (mmHg)	Treatment Goal
Normal	<120	<80	Maintain lifestyle habits
Elevated	120–129	-	Lifestyle modification
Stage 1 Hypertension	130–139	80–89	Goal SBP <130; consider <120 if tolerated
Stage 2 Hypertension	≥140	≥90	Goal SBP <130; consider <120 if tolerated

The 2025 AHA/ACC guideline acknowledges RDN as an emerging therapeutic option for patients with uncontrolled or resistant hypertension, emphasizing its role within a comprehensive, individualized management strategy. Multidisciplinary care, integrating hypertension specialists, trained proceduralists, and primary care clinicians, is essential to ensure appropriate candidate evaluation, procedural safety, and individualized risk‑benefit assessment. Shared decision‑making remains central, incorporating patient preferences, comorbidities, and treatment goals. While further research is needed, RDN represents a promising adjunctive strategy for patients with persistently uncontrolled blood pressure despite optimal medical therapy to prevent major adverse cardiovascular events and all-cause mortality [10].

Physiological background and devices

Knowledge of the role of renal sympathetic nerves in blood pressure control dates to the mid‑19th century. Recent studies using direct electrical stimulation or complete denervation have clarified the physiological impact of altered renal sympathetic activity [4]. The effect on renal function and blood pressure depends on the sympathetic activation level: low levels stimulate renin release; moderate levels increase sodium reabsorption; high levels elevate renal vascular resistance.

Informed by these mechanisms, three percutaneous RDN methods have emerged, as shown in Table 3.

**Table 3:** Main devices currently in use Source: [11]

The three main devices currently studied
a) Symplicity Spyral (Medtronic): Four‑electrode spiral RF catheter delivering medium‑frequency alternating current to periadventitial nerves while sparing the arterial wall.
b) Paradise (Recor Medical): Delivers ultrasound energy via an inflatable balloon catheter that simultaneously circulates cooling solution in the vessel lumen, allowing safe ablation.
c) Peregrine (Ablative Solutions): Uses ethanol delivered by concentric micro‑needles inserted through the arterial wall into the perivascular space to chemically ablate nerves.

These approvals followed strong evidence from sham‑controlled RCTs (e.g., RADIANCE‑HTN SOLO, TRIO; SPYRAL HTN-OFF MED and ON MED) which demonstrated modest but consistent reductions in blood pressure (on the order of ~5-10 mm Hg systolic), combined with a favorable safety profile and no significant increases in major adverse events when compared to sham controls. Professional society coverage (e.g., SCAI, AHA) and expert commentary support the approval as a game-changing but supplemental option in the management of uncontrolled hypertension in selected candidate patients [12-16].

Safety

RDN is performed via femoral access (radial approaches are in development) and typically lasts around an hour. Beyond standard femoral access risks (bleeding, infection, arterial dissection, thromboembolism, hematoma, pseudoaneurysm, arteriovenous fistula), contrast usage and radiation exposure are considerations. Analgesics are administered to minimize pain.

Immediate safety data from multiple RCTs and meta‑analyses (2013-2022) found no significant difference in major adverse events versus controls [17-19]. The most common minor complication was lingering pain over two days (~12% incidence); serious complications (access‑site events, renal artery dissection, death) had an incidence of <1%. Protocols do not routinely require post‑procedural imaging, though Doppler ultrasound, CT angiography, or MR angiography may be considered clinically to assess for renal artery stenosis or dissection.

Long‑term safety data show a low incidence of major events beyond 30 days. Adverse clinical events are more likely in patients with higher cardiovascular risk. Renal artery stenosis post‑RF ablation remains a concern, with an estimated intervention‐requiring incidence of 0.2% per year; however, similar to the natural occurrence rate in hypertensive patients, the highest risk of cardiovascular complications lies within the first 6-month period.

Importantly, renal function remains unaffected post‑RDN, even in patients with moderate-severe chronic kidney disease or systolic heart failure. A three‑year analysis from the Global SYMPLICITY Registry showed no late safety concerns [20].

Limitations and future directions

Most patients experience sustained modest blood pressure reductions; however, some have partial responses or revert to baseline over time. A theoretical concern is sympathetic nerve re‐innervation, which may lessen the procedure’s effect. Animal studies showing partial or complete nerve regrowth still demonstrated persistent blood pressure reductions, raising questions about functional re‐innervation. Factors negatively influencing response include lower baseline blood pressure, increased arterial stiffness, and anatomical variants (e.g., accessory arteries). Blood pressure control over time may vary due to aging, comorbidities, medication changes, or nerve regrowth.

In conclusion, RDN has an immediately favorable safety profile with low major adverse event rates. Medium‑ and long‑term safety data indicate a continued absence of serious complications and no renal impairment or significant stenosis. Further research is required to understand re‑innervation in humans and to address long‑term blood pressure increase post‑RDN.

## Conclusions

Renal denervation was well-tolerated in our patient, with early post‑procedure improvement in blood pressure by day 3. This early stabilization was followed by sustained blood pressure control at the three‑month follow‑up, without significant antihypertensive medication adjustments, during which time she had improved quality of life and no emergency department visits due to a hypertensive urgency or hypertensive emergency with target organ damage.
